# Diagnostic yield of exome sequencing for prenatal diagnosis of fetal structural anomalies: A systematic review and meta‐analysis

**DOI:** 10.1002/pd.6115

**Published:** 2022-05-07

**Authors:** Rhiannon Mellis, Kathryn Oprych, Elizabeth Scotchman, Melissa Hill, Lyn S Chitty

**Affiliations:** ^1^ North Thames Genomic Laboratory Hub Great Ormond Street Hospital for Children NHS Foundation Trust London UK; ^2^ Genetics and Genomic Medicine UCL Great Ormond Street Institute of Child Health London UK; ^3^ South West Thames Regional Genetics Service London UK

## Abstract

**Objectives:**

We conducted a systematic review and meta‐analysis to determine the diagnostic yield of exome sequencing (ES) for prenatal diagnosis of fetal structural anomalies, where karyotype/chromosomal microarray (CMA) is normal.

**Methods:**

Following electronic searches of four databases, we included studies with ≥10 structurally abnormal fetuses undergoing ES or whole genome sequencing. The incremental diagnostic yield of ES over CMA/karyotype was calculated and pooled in a meta‐analysis. Sub‐group analyses investigated effects of case selection and fetal phenotype on diagnostic yield.

**Results:**

We identified 72 reports from 66 studies, representing 4350 fetuses. The pooled incremental yield of ES was 31% (95% confidence interval (CI) 26%–36%, *p* < 0.0001). Diagnostic yield was significantly higher for cases pre‐selected for likelihood of monogenic aetiology compared to unselected cases (42% vs. 15%, *p* < 0.0001). Diagnostic yield differed significantly between phenotypic sub‐groups, ranging from 53% (95% CI 42%–63%, *p* < 0.0001) for isolated skeletal abnormalities, to 2% (95% CI 0%–5%, *p* = 0.04) for isolated increased nuchal translucency.

**Conclusion:**

Prenatal ES provides a diagnosis in an additional 31% of structurally abnormal fetuses when CMA/karyotype is non‐diagnostic. The expected diagnostic yield depends on the body system(s) affected and can be optimised by pre‐selection of cases following multi‐disciplinary review to determine that a monogenic cause is likely.

## Key points

What's already known about this topic?

- Prenatal exome sequencing (ES) increases genetic diagnoses in fetuses with structural abnormalities and a normal karyotype and chromosomal microarray.
- Published diagnostic yields from ES are varied and may be influenced by study size, case selection and fetal phenotype.

What does this study add?

- This study provides a comprehensive systematic review of the literature to date and investigates the diagnostic yield of ES for a range of isolated system anomalies, to support clinical decision‐making on how to offer prenatal ES.

## 1 INTRODUCTION

Ultrasound scanning in pregnancy can reveal fetal structural abnormalities from the first trimester onwards,^1^ ranging from isolated minor anomalies to severe, multisystem disorders. These abnormalities may indicate an underlying genetic condition but making a definitive diagnosis can be difficult because many conditions have overlapping fetal presentations. A genetic diagnosis can clarify the prognosis for the fetus, informing parental decision making and clinical management during pregnancy and the perinatal period.

Conventional genetic testing for fetal anomalies comprises quantitative fluorescence PCR (QF‐PCR) for rapid aneuploidy exclusion, and karyotype or chromosomal microarray (CMA) for detection of chromosomal and sub‐chromosomal abnormalities. Together, these can provide a diagnosis in up to 40% of cases^2^, ^3^, ^4^, but leave the majority undiagnosed. Targeted sequencing of individual genes can be offered in cases where clinical features or family history suggest a specific monogenic disorder but genome‐wide next generation sequencing (NGS) is now being used more frequently to allow interrogation of multiple genes in a single test. Exome sequencing (ES) examines the 1%–2% of the genome that encodes proteins and harbours around 85% of known disease‐causing genetic variants, and is generally less costly and easier to interpret than whole genome sequencing (WGS). ES can be applied in practice as whole ES (sequencing or analysing all exons), medical/clinical ES (sequencing or analysing only the exons of known Mendelian disease genes), or targeted ES (applying ‘virtual’ gene panels to ES data to limit analysis to genes of interest for a specific phenotype).

In the postnatal setting ES has proven a powerful tool for the diagnosis of children with neurodevelopmental delay but its application in prenatal diagnosis initially lagged behind. This is largely because long turnaround times precluded the return of results within the timeframe of a pregnancy. Additionally, incomplete or unrecognised fetal phenotypes complicate the interpretation of genetic variants from prenatal ES. Over recent years, rapid analytical pipelines have made it possible to return ES results during an ongoing pregnancy and it is now considered for prenatal diagnosis of fetal structural anomalies in the guidance of several professional bodies.^9^, ^10^, ^11^ Two large prospective studies demonstrated the diagnostic utility of ES for prenatal diagnosis of unselected fetal structural abnormalities, reporting diagnostic rates of 8.5%–10%,^12^ ^,^ ^13^ while numerous smaller studies report diagnostic rates above 80% in highly‐selected case series.^7^ ^,^ ^14^ ^,^ ^15^ This body of literature has been previously reviewed^4^ ^,^ ^16^ ^,^ ^17^ but has rapidly expanded over the last 2 years.

As prenatal ES is increasingly adopted in clinical settings there remain important questions around best practice for implementation. It will be of interest to health professionals and policy makers to define expected diagnostic yields from prenatal ES for different fetal presentations. This information may help to manage parental expectations during pre‐test counselling and will inform development of further guidelines on when and how to offer prenatal ES. In this systematic review and meta‐analysis we aim to address the question ‘what is the diagnostic yield of ES for prenatal diagnosis in pregnancies complicated by fetal structural anomalies where fetal karyotype/microarray is normal?’ We also explore the diagnostic yield in different phenotypic sub‐groups.

## 2 METHODS

### 2.1 Protocol and registration

We devised a systematic review protocol in line with PRISMA guidance^18^ ^,^ ^19^ and registered it prospectively on the PROSPERO international register of systematic reviews (reference CRD42020200600). Study authors agreed the protocol prior to conducting the searches. Any required small amendments were made with the consensus of all authors.

### 2.2 Eligibility criteria

Studies were included in this review if they met the following criteria: (i) Retrospective or prospective cohorts of 10 or more pregnancies undergoing ES (whole, clinical or targeted) or WGS for diagnosis of fetal structural anomalies, (ii) CMA/karyotype was negative or non‐diagnostic, (iii) Testing was initiated based on the prenatal phenotype, (iv) Full text report was available in English language.

The focus of this review is prenatal diagnosis of structural anomalies, therefore studies with mixed pre‐ and postnatal presentations were only included if it was possible to extract the prenatal cases. Similarly, for studies with mixed indications for testing (e.g. some cases initiated because of fetal phenotype and some because of family history) we only included studies where fetal anomaly cases could be extracted. Studies where ES was completed postnatally for some or all cases but had been initiated based on the prenatal phenotype alone were included. Only variants classified as pathogenic/likely pathogenic according to diagnostic‐standard guidelines *and* determined to be causative of the fetal phenotype were considered in diagnostic yield, therefore studies using ES purely for novel candidate gene discovery were not included. We also excluded case reports, series of fewer than 10 cases, reviews, editorials and commentaries, and studies using gene panel testing rather than ES.

### 2.3 Information sources and search strategy

Electronic searches of four databases (MEDLINE, Embase, Cochrane library, and Web of Science) were conducted for records published between January 2010 and October 2021. This date range was chosen because prenatal ES was not used prior to 2010. The search was initially conducted on 27/08/2020 and repeated on 15/10/2021. Additional potentially relevant studies were identified by manually searching reference lists of relevant studies and published reviews, and conference proceedings of prenatal and genetics conferences of relevant major societies in last 3 years.

Search terms were variations on the keywords ‘prenatal diagnosis’ and ‘exome sequencing’. Alternative terms for ‘prenatal diagnosis’ included ‘fetal’, ‘fetus’, ‘prenatal’, and ‘antenatal’. Alternative terms for ‘exome sequencing’ included ‘whole exome sequencing’, ‘exome’, ‘whole genome sequencing’, ‘genome, human’ and ‘sequence analysis, DNA’. The full search strategy is detailed in supporting information S1. We chose relatively broad search terms in an effort to capture as many relevant studies as possible; for the same reason no search limits were applied apart from date range.

### 2.4 Study selection

After the removal of duplicates, two reviewers (RM and KO) independently screened titles and abstracts. For abstracts identified as potentially relevant, full text articles were retrieved and reviewed against the inclusion and exclusion criteria. Any disagreement between reviewers was resolved by discussion. Where the same data were presented more than once, the most recent study was selected.

### 2.5 Data extraction

The following data, where available, were extracted by two reviewers (RM and ES) into a datasheet: study setting, sample size, study inclusion criteria, ES approach, phenotypes used for interpretation (prenatal only or prenatal plus postnatal/post‐mortem), number of fetuses with diagnostic variants, variants of uncertain significance (VUS), incidental findings, gestation at testing, test turnaround time, pregnancy outcomes, and impact on management. For studies that performed CMA in parallel with sequencing, the cases with negative CMA were extracted, in order to be comparable with other studies where chromosomal abnormalities were ruled out prior to ES.

### 2.6 Quality assessment of included studies

The quality of the studies was assessed using a modified Standards for Reporting of Diagnostic Accuracy (STARD) checklist,^20^ comprising 22 items. The items for reporting considered most important for these studies (as they may influence the reported diagnostic yield) were: (i) clearly described selection criteria, (ii) whether diagnostic variants were confirmed by Sanger sequencing, (iii) whether American College of Medical Genetics and Genomics (ACMG) or equivalent standardised classification was used for variant interpretation, (iv) approach to analysing and reporting VUS and incidental findings addressed. For eight studies the assessment was done independently by two reviewers (RM and MH) with discrepancies resolved by discussion. Once consensus was reached on how to apply the criteria and reviewers' scores were concordant on new study assessments, the remaining studies were assessed by RM only.

### 2.7 Data synthesis

The primary outcome of interest was the incremental diagnostic yield of ES (i.e. the proportion of cases in which a diagnostic genetic variant is detected by ES) over CMA/karyotype, expressed as a risk difference. This was calculated for each study with 95% confidence intervals (CI) and pooled for all studies in a meta‐analysis, using a random effects model with inverse variance weighting. A subgroup analysis was performed to investigate the effect on diagnostic yield of pre‐test case selection for higher likelihood of monogenic disease, where this information was available. Where studies reported data on isolated system anomalies classified by different phenotypic groups, a further subgroup analysis was performed to estimate pooled incremental yields by phenotypic group. Only phenotype groups reported in at least three studies were included in this analysis. Findings were displayed using forest plots. Between‐study heterogeneity was assessed visually and using an I^2^ statistic with a Q‐test. Publication bias was assessed graphically using a funnel plot with Egger's test. All statistical analyses were performed in R studio^21^ v1.3.1093 using the ‘metafor’ package.^22^

## 3 RESULTS

**FIGURE 1:**
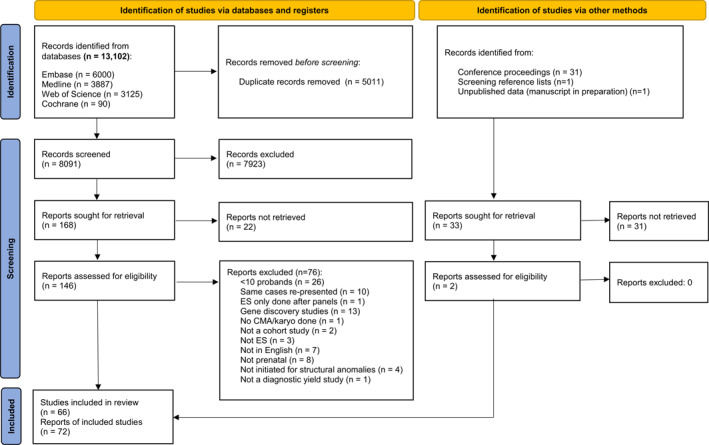
PRISMA flow diagram showing study screening and selection

Database searches identified 8123 unique records in total. After screening by title and abstract, and retrieving full text reports, 148 articles were assessed, of which 72 reports from 66 studies were deemed eligible and included in the final review (Figure 1, Table 1). Six reports examined specific phenotypic sub‐groups from previously published larger cohorts,^23^, ^24^, ^25^, ^26^, ^27^, ^28^ hence were not separate studies. These were excluded from the overall meta‐analysis of incremental yield and the subgroup analysis by case selection, to avoid double‐counting of cases. However, these reports provide updated information in their respective phenotypic groups so were included in the subgroup analysis by phenotypic group, in place of the previously reported sub‐groups from their parent studies. Across 66 studies, this review represents 4350 probands undergoing prenatal ES.

**TABLE 1:** Characteristics and results of 72 prenatal ES study reports included in the systematic review

Study	No. of cases with normal CMA	Inclusion criteria	Sequencing and analysis approach	Diagnostic variants (%)	VUS or candidate genes (%)	Incidental findings (%)	Clinical impact (%)	Average TAT in days (range, if given)
Carss et al, 2014^a^	28	Fetuses and neonates with prenatal structural anomalies	Trio WES	3/28 (11%)	5/28 (18%)	ND	ND	ND
Drury et al, 2015^a^	24	Fetuses with structural abnormalities, and/or increased NT (>3.5 mm)	Solo or Trio CES	5/24 (21%)	1/24 (4%)	2/24 (8%)	ND	ND
Pangalos et al, 2016	14	Fetuses with (mild or severe) structural anomalies	Solo TES (758 prenatal disorders gene panel)	6/14 (43%)	1/14 (7%)	0/14	ND	<10
Yates et al, 2017	84	Fetuses with US anomalies resulting in TOP or IUFD	Solo or Trio WES	17/84 (20%)	45/84 (54%)	2/84 (2%)	ND	ND
Aarabi et al, 2018^a^	20	≥1 major structural defect detected prenatally	Trio WES or CES	1/20 (5%)	2/20 (10%)	ND	ND	ND
Armes et al, 2018^a^	16	Fetal or early neonatal death, having undergone full autopsy, genetic aetiology suspected (PM)	Trio WGS with panel analysis	2/16 (13%)	6/16 (38%)	ND	ND	ND
Boissel et al, 2018^a^ ^,^ ^b^	101	Fetuses or stillborns with severe anomalies:	Solo or Trio WES	19/101 (19%)	7/101 (7%)	ND	ND	ND
Chandler et al, 2018^a^ ^,^ ^b^	16	Fetuses with suspected skeletal dysplasia on prenatal US	Trio TES (240 skeletal gene panel)	13/16 (81%)	2/16 (13%)	ND	NQ	16.5 (11–41)
Fu et al, 2018^a^ ^,^ ^b^	196	Fetuses with ultrasound detected structural malformations, excluding isolated increased NT ≥ 3.5 mm/cystic hygroma, and isolated soft markers	Solo or Trio CES	47/196 (24%)	25/196 (13%)	12/196 (6%)	ND	∼21
Leung et al, 2018^a^	33	Fetuses with structural anomalies detected on prenatal US	Trio WES	3/33 (9%)	6/33 (18%)	ND	ND	ND
Shamseldin et al, 2018^a^	44	Severe fetal malformations, lethal NIHF or unexplained IUFD	Solo WES	22/44 (50%)	15/44 (34%)	ND	ND	ND
Zhou, X et al, 2018^a^ ^,^ ^b^	12	Fetuses with suspected skeletal dysplasia on US	Solo TES (363 skeletal dysplasia gene panel)	10/12 (83%)	ND	ND	ND	42
Choy et al, 2019^a^ ^,^ ^b^	42	Fetuses with increased NT ≥ 3.5 mm +/− structural anomalies	Solo WGS	7/42 (17%)	18/42 (43%)	ND	None ‐ results not returned to families	10
Daum et al, 2019^a^ ^,^ ^b^	77	Fetuses with US‐detected malformations OR family history suggestive of a genetic diagnosis	Solo or Trio WES	16/77 (21%)	ND	ND	NQ	26 (19–33)
De Koning et al, 2019^a^	20	Fetuses with one or more structural anomalies, genetic aetiology suspected	Trio WES	8/20 (40%)	1/20 (5%)	3/20 (15%)	9/12 (75%)	10.5 (7–19)
Greenbaum et al, 2019	45	Fetuses with abnormal US findings and/or relevant family history	Solo or Trio WES	13/45 (29%)	ND	ND	ND	Ongoing pregnancies: 15–20
								Post‐mortem: 15–60
Liu et al, 2019^a^ ^,^ ^b^	28	Fetuses with skeletal abnormalities on US	Solo TES (363 skeletal dysplasia gene panel)	16/28 (57%)	5/28 (18%)	ND	NQ	ND
Lord et al, 2019^a^ ^,^ ^b^	610	Fetuses with any structural anomaly on US, including isolated increased NT ≥ 4 mm	Trio TES (1628 developmental disorder gene panel)	52/610 (9%)	24/610 (4%)	ND	NQ	ND
Meier et al, 2019^a^	19	Severe fetal anomalies identified by US scan: (i) Two or more anomalies associated with a high risk for fetal or perinatal lethality and suspected genetic aetiology, or (ii) familial recurrence of the fetal anomaly phenotype	Trio WES	6/19 (32%)	6/19 (32%)	ND	ND	ND
Normand et al, 2019^a^	146	Fetuses with at least one structural anomaly detected by prenatal imaging or autopsy	Solo or Trio WES	46/146 (32%)	ND	3/62 (5%)	15/19 (79%)	Rapid: 14 (7–38)
								Trio: 43 (13–78)
								Solo: 88 (18–141)
Petrovski et al, 2019^a^ ^,^ ^b^	234	Any ultrasound identified fetal structural anomalies, including isolated NT ≥3·5 mm	Trio WES	24/234 (10%)	46/234 (20%)	4/234 (2%)	NQ	28–56
Quinlan‐Jones et al, 2019^a^	27	Fetuses with a significant structural anomaly resulting in TOP, IUFD, or NND	Trio TES (1628 developmental disorder gene panel)	10/27 (37%)	6/27 (22%)	ND	NQ	ND
Westphal et al, 2019^a^ ^,^ ^b^	30	Fetuses and neonates with prenatally diagnosed CHDs (focus on severe and syndromic)	Trio WES	6/30 (20%)	4/30 (13%)	3/30 (10%)	ND	ND
Aggarwal et al, 2020^a^	32	Fetuses with structural abnormalities or unexplained IUFD, genetic aetiology suspected (PM)	Solo and Trio, WES or CES	18/32 (56%)	4/32 (13%)	ND	ND	ND
Becher et al, 2020^a^	35	One or more fetal malformations or severe fetal hydrops, genetic aetiology suspected	Trio WES	9/35 (26%)	7/35 (20%)	1/35 (3%)	NQ	Urgent: 12 (9–16)
								Non‐urgent: 120–180
Chen et al, 2020^a^ ^,^ ^b^	105	Fetuses with structural anomalies, including isolated increased NT >3.5mm	Trio CES	20/105 (19%)	12/105 (11%)	ND	ND	25
Corsten‐Janssen et al, 2020^a^	23	Fetuses with major structural anomalies	Trio TES (∼3850 OMIM disease gene panel with late onset diseases excluded)	8/23 (35%)	ND	11/23 (48%)	6/23 (26%)	14 (8–20)
Deden et al, 2020^a^ ^,^ ^b^	54	Fetuses with multiple congenital anomalies. Isolated major anomaly or multiple soft markers only if high suspicion of genetic aetiology	Trio WES	18/54 (33%)	2/54 (4%) both later re‐classified as pathogenic	ND	24/37 (68%)	10 (4–28)
Deng et al, 2020^a^ ^,^ ^b^	21	Fetuses with NIHF +/‐ other structural anomalies	Trio CES	3/21 (14%)	2/21 (10%)	ND	NQ	ND
Guo et al, 2020^a^	40	Recurrent fetal congenital abnormalities or dysmorphic features	Trio WES	12/40 (30%)	20/40 (50%)	ND	NQ	ND
Han et al, 2020^a^ ^,^ ^b^	26	Fetal anomalies suggestive of a skeletal dysplasia	Trio CES	23/26 (89%)	1/26 (4%)	1/26 (4%)	NQ	<14
Heide et al, 2020^a^ ^,^ ^b^	62	Fetuses with abnormal corpus callosum (complete or partial agenesis, or short CC) detected on US +/‐ other anomalies	Trio TES (425 brain anomaly gene panel)	Or 12/62 (19%)	6/65 (9%)	ND	NQ	21.5 (9–53)
Lefebvre et al, 2020^a^	95	Fetuses with multiple congenital anomalies	Solo WES	24/95 (25%)	14/95 (15%)	ND	ND	ND
Lei et al, 2020^a^ ^,^ ^b^	163	Fetuses with CAKUT +/‐ other structural anomalies	Trio CES	20/163 (12%)	2/163 (1%)	9/163 (6%)	NQ	14–84
Li, L et al, 2020^a^ ^,^ ^b^	19	Fetuses with cerebellar vermis defects +/‐ other structural anomalies	Solo or Trio CES	8/19 (42%)	2/19 (11%)	1/19 (5%)	ND	28–56
Li, R et al, 2020^a^ ^,^ ^b^	260	Fetuses with congenital heart defects +/‐ other structural anomalies	Trio CES	26/260 (10%)	16/260 (6%)	7/260 (3%)	ND	21–56
Mone et al, 2020^b^ ^,^ ^c^	197	Fetuses with prenatally detected congenital heart disease (excluding small muscular VSDs), extracted from the PAGE study extended cohort (Lord et al, 2019)	Trio TES (1628 developmental disorder gene panel)	25/197 (13%)	10/197 (5%)	ND	ND	ND
Pooh et al, 2020^a^	16	Fetuses with anomalies detected on US at 11‐13 weeks, genetic disease strongly suspected	Trio CES	7/16 (44%)	ND	ND	ND	7–10
Reischer et al, 2020^a^ ^,^ ^b^	11	Fetuses with decreased/absent fetal movement plus arthrogryposis of limbs	Solo WES	6/11 (55%)	1/11 (9%)	ND	ND	ND
Sparks et al, 2020^a^ ^,^ ^b^	127	Fetuses with NIHF, fetal ascites, pleural or pericardial effusions, skin oedema, cystic hygroma, increased NT, or a combination of these conditions	Trio WES	37/127 (29%)	12/127 (9%)	4/115	NQ	Ongoing pregnancies: 14–28
								Post‐mortem: 56–84
Sun et al, 2020^a^ ^,^ ^b^	66	Fetuses with cardiac left‐sided lesions	Solo or Trio WES	13/66 (20%)	11/66 (17%)	1/66 (2%)	ND	ND
Vora et al, 2020^a^	102	Fetuses with isolared or multiple congenital anomalies	Trio WES (*n* = 99) or WGS (*n* = 3)	21/102 (21%)	10/102 (10%) 2 re‐classified as benign after 5 yrs	9/204 (4%) in consenting parents	NQ	182–365
Xue et al, 2020^a^ ^,^ ^b^	24	Fetuses with increased NT and no detectable structural malformations on US	Trio WES	3/24 (13%)	2/24 (8%)	ND	ND	ND
Yang et al, 2020^a^ ^,^ ^b^	73	Fetuses with isolated first‐trimester increased NT ≥3.5 mm	Trio CES	4/73 (6%)	7/73 (10%)	ND	ND	ND
Zhou, X et al, 2020^a^ ^,^ ^b^	41	Fetuses with isolated renal abnormalities on US	Solo or Trio WES	3/41 (7%)	ND	1/41 (2%)	NQ	ND
Qi et al, 2020^a^ ^,^ ^b^	80	Fetuses with at least one ultrasonographic structural anomaly, including isolated increased NT	Trio CES (5000 OMIM genes) +/‐ WES (where sufficient DNA)	27/80 (33.8%)	5/80 (6.3%)	ND	ND	14–28
Rinaldi et al, 2020^a^	30	Fetuses with structural malformation or ‘severe condition’ on US (e.g. growth restriction, absence of spontaneous movements)	Trio CES (5817 OMIM genes)	19/30 (63%)	0/30	ND	None in affected pregnancy ‐ sequenced after end of pregnancy	ND
Al‐Kouatly et al, 2021^a^	22	Fetuses with confirmed NIHF after first trimester +/‐ other structural anomalies (strict phenotypic description for NIHF: at least two abnormal fluid collections)	Trio CES	11/22 (50%)	5/22 (22.7%)	ND	NQ	14–21
Aoi et al, 2021^a^	17	Fetuses with ultrasonographic structural anomalies	Solo or Trio WES	5/17 (29.4%)	1/17 (5.9%)	ND	ND	ND
Cao et al, 2021^a^ ^,^ ^b^	11	Fetuses with abnormalities suggestive of fetal akinesia	Trio CES (4200 OMIM genes)	5/11 (45.5%)	3/11 (27.3%)	ND	None in affected pregnancy ‐ sequenced after end of pregnancy	ND
Correa et al, 2021^a^ ^,^ ^b^	19	Fetuses with confirmed NIHF after first trimester +/‐ other structural anomalies (strict phenotypic description for NIHF: at least two abnormal fluid collections)	Solo WES	7/19 (36.8%)	1/19 (5.3%)	ND	ND	ND
De Koning et al, 2021^b^ ^,^ ^c^	18	Fetuses with CNS malformations detected by prenatal US; isolated or with other structural anomalies. (Some cases previously published in De Koning et al, 2019)	Trio WES	10/18 (56%)	2/18 (11.1%)	2/18 (11.1%)	12/18 (67%)	ND
Dempsey et al, 2021^a^	52	Ongoing pregnancies with US‐detected fetal structural abnormalities where MDT judged a high likelihood of phenotype being explained by a single gene disorder, and where the result may influence management of the pregnancy, labour or early neonatal care	Trio WES with phenotype‐specific panel analysis	19/52 (37%)	13/52 (25%)	ND	17/43 (39.5%) in pregnancies ongoing at time of result	17
He et al, 2021^b^	94	Fetuses with structural anomalies detected by prenatal US in the second trimester	Solo or Trio WES	37/94 (39.4%)	14/94 (14.9%)	4/94 (4.3%)	ND	42–56
Kucińska‐Chahwan et al, 2021^a^ ^,^ ^b^	26	Fetuses with skeletal abnormalities, excluding isolated talipes	Solo WES	18/26 (69.2%)	ND	ND	ND	84
Lei et al, 2021^a^ ^,^ ^b^	85	Fetuses with US‐detected structural anomalies, other than skeletal abnormalities	Solo WES	16/85 (18.8%)	16/85 (18.8%)	ND	ND	14–21
Liao et al, 2021^b^	24	Fetuses with ultrasonographic diagnosis of abnormal sylvian fissure	Solo, Duo or Trio WGS	12/24 (50%)	ND	ND	ND	ND
Mellis et al, 2021^b^ ^,^ ^c^	213	Fetuses with increased NT ≥3.5 mm at 11–14 weeks of gestation +/‐ other anomalies, extracted from the PAGE and Columbia studies' extended cohorts (Lord et al, 2019; Petrovski et al, 2019)	Trio TES (1628 developmental disorder gene panel) or WES	28/213 (13.1%)	8/213 (3.8%)	ND	ND	ND
Mone et al, 2021^b^ ^,^ ^c^	28	Fetuses with NIHF +/‐ other anomalies, extracted from the PAGE study extended cohort (Lord et al, 2019)	Trio TES (1628 developmental disorder gene panel)	7/28 (25%)	2/28 (7.1%)	ND	ND	ND
Peng et al, 2021^a^ ^,^ ^b^	38	Fetuses with suspected skeletal dysplasia on prenatal US	Solo or Trio WES	25/38 (65.8%)	4/38 (10.5%)	12/38 (31.6%)	ND	ND
Qiao et al, 2021^a^ ^,^ ^b^	300	Fetuses with CHD detected by echocardiography	Solo or Trio WES	24/300 (8%)	32/300 (10.7%)	incidental: 37/300 (12.3%)	ND	ND
						secondary “looked for”: 11/300 (3.7%)		
Sun et al, 2021^a^ ^,^ ^b^	19	Fetuses with non‐compaction cardiomyopathy on fetal echocardiogram	Trio WES	8/19 (42.1%)	8/19 (42.1%)	ND	ND	ND
Tang et al, 2021^a^ ^,^ ^b^	15	Fetuses with suspected skeletal dysplasias on US scanning	Trio WES	10/15 (66.7%)	1/15 (6.7%)	ND	ND	ND
Tolusso et al, 2021^a^	20	Fetuses with congenital anomalies judged likely to be caused by an underlying monogenic condition	Trio WES	8/20 (40%)	7/20 (35%)	1/20 (5%)	9/20 (45%)	Ongoing pregnancies: 19.3
								Terminated pregnancies: 56.8
Wagner et al, 2021^b^ ^,^ ^c^	15	Fetuses with NIHF detected on US. (Some cases previously published in Daum et al, 2019)	Trio WES	7/15 (47%)	ND	ND	ND	ND
Zhang, F et al, 2021^b^	12	Fetuses with isolated absent or hypoplastic nasal bone	Solo CES (2742 OMIM genes)	2/12 (16.7%)	ND	ND	ND	ND
Zhang, L et al, 2021^a^ ^,^ ^b^	55	Fetuses with suspected skeletal dysplasias based on US features	Solo or Trio WES	35/55 (64%)	4/55 (7.3%)	ND	ND	21–70
Zhang, X et al, 2021^a^ ^,^ ^b^	27	Fetuses with suspected skeletal dysplasias undergoing ToP before the third trimester	Trio WES (*n* = 17) or TES (*n* = 10 (505 skeletal dysplasia gene panel))	21/27 (77.8%)	ND	ND	ND	∼28
Zhen et al, 2021^b^	13	Fetuses with apparently isolated micrognathia identified on US at 11–14 weeks	Trio CES (4200 OMIM disease genes)	8/13 (61.5%)	ND	ND	ND	ND
Zhou, J et al, 2021^a^ ^,^ ^b^	102	Fetuses with structural or growth anomalies identified by US scanning	Trios WES and WGS in parallel	13/102 (12.7%)	ND	ND	NQ	21 (15–27)
Zhou, X et al, 2021^a^ ^,^ ^b^	28	Fetuses with recurrent NIHF +/− additional structural anomalies, where immune, infectious causes or thalassaemia ruled out	Trio WES	10/28 (36%)	5/28 (18%)	ND	ND	28–42
Baptiste et al, 2022^b^ ^,^ ^c^	268	Fetuses with CNS abnormalities +/− other anomalies, extracted from the PAGE and Columbia studies' extended cohorts (Lord et al, 2019; Petrovski et al, 2019)	Trio TES (1628 developmental disorder gene panel) or WES	36/268 (13.4%)	11/268 (4.1%)	ND	ND	ND

Abbreviations: CES, clinical exome sequencing; CHD, congenital heart defect, IUFD, intrauterine fetal demise, MDT, multi‐disciplinary team; ND, not described; NIHF, non‐immune hydrops fetalis; NND, neonatal death; NQ, not quantified; NT, nuchal translucency; OMIM, Online Mendelian Inheritance in Man; PM, post‐mortem; TAT, turnaround time; TES, targeted exome sequencing; TOP, termination of pregnancy; US, ultrasound; VUS, variants of unknown significance; WES, whole exome sequencing.

^a^ studies included in the case selection subgroup analysis (those which it was possible to determine whether cases were selected based on higher likelihood of monogenic aetiology, and where all cases in the cohort were subject to these selection criteria).

^b^ studies included in the phenotype subgroup analysis (those which recruited a single phenotypic group or reported breakdown of cases by phenotypic group/affected body system and distinguished single system anomalies from multisystem).

^c^ reports of phenotypic sub‐groups from previously published larger cohorts, where cases overlap with previous publication.

### 3.1 Study characteristics

**FIGURE 2:**
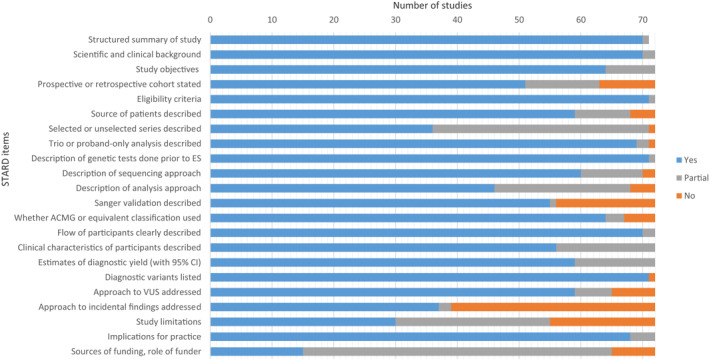
Quality assessment of included study reports using modified Standards for Reporting of Diagnostic Accuracy (STARD) criteria. (*n* = 72, except or the item “structured summary of study”, where *n* = 71 because one report was a Letter to the Editor, which would not be expected to contain a structured abstract)

The characteristics of the included studies are summarised in Table 1. Of the 72 included reports, 48 (67%) were published in 2020‐21. Three studies used WGS rather than ES, with or without applying phenotype‐specific panels for analysis.^29^, ^30^, ^31^ Eighteen studies used a clinical exome sequencing (CES) approach in some or all cases, which here we define as targeting either the sequencing or the analysis to Online Mendelian Inheritance in Man disease genes.^14^ ^,^ ^32^ ^,^ ^41^, ^42^, ^43^, ^44^, ^45^, ^46^, ^47^, ^48^ ^,^ ^33^, ^34^, ^35^, ^36^, ^37^, ^38^, ^39^, ^40^ Eight studies targeted ES analysis more tightly using phenotype‐specific gene panels,^7^ ^,^ ^12^ ^,^ ^15^ ^,^ ^49^, ^50^, ^51^, ^52^, ^53^ while the remainder took a whole ES approach.^8^ ^,^ ^13^ ^,^ ^62^, ^63^, ^64^, ^65^, ^66^, ^67^, ^68^, ^69^, ^70^, ^71^ ^,^ ^54^ ^,^ ^72^, ^73^, ^74^, ^75^, ^76^, ^77^, ^78^, ^79^, ^80^, ^81^ ^,^ ^55^ ^,^ ^82^, ^83^, ^84^, ^85^, ^86^, ^87^, ^88^ ^,^ ^56^, ^57^, ^58^, ^59^, ^60^, ^61^ Where stated (15 studies),^7^ ^,^ ^8^ ^,^ ^74^ ^,^ ^75^ ^,^ ^78^ ^,^ ^82^ ^,^ ^84^ ^,^ ^15^ ^,^ ^29^ ^,^ ^36^ ^,^ ^52^ ^,^ ^53^ ^,^ ^55^ ^,^ ^62^ ^,^ ^73^ the median turnaround time for ES was 20 days (range 4–141). Studies included fetuses with a range of structural abnormality phenotypes, with some studies recruiting cases with fetal anomalies in a specific body system (e.g. renal anomalies or cardiac defects) and others including any structural anomaly. In total, 42/66 studies recruited cases with anomalies in a specific body system or broke down fetal abnormalities within the cohort into distinct phenotypic groups (Table 1). The quality of included reports, assessed using modified STARD criteria, was generally high (Figure 2).

### 3.2 Incremental yield of diagnostic variants from ES

**FIGURE 3:**
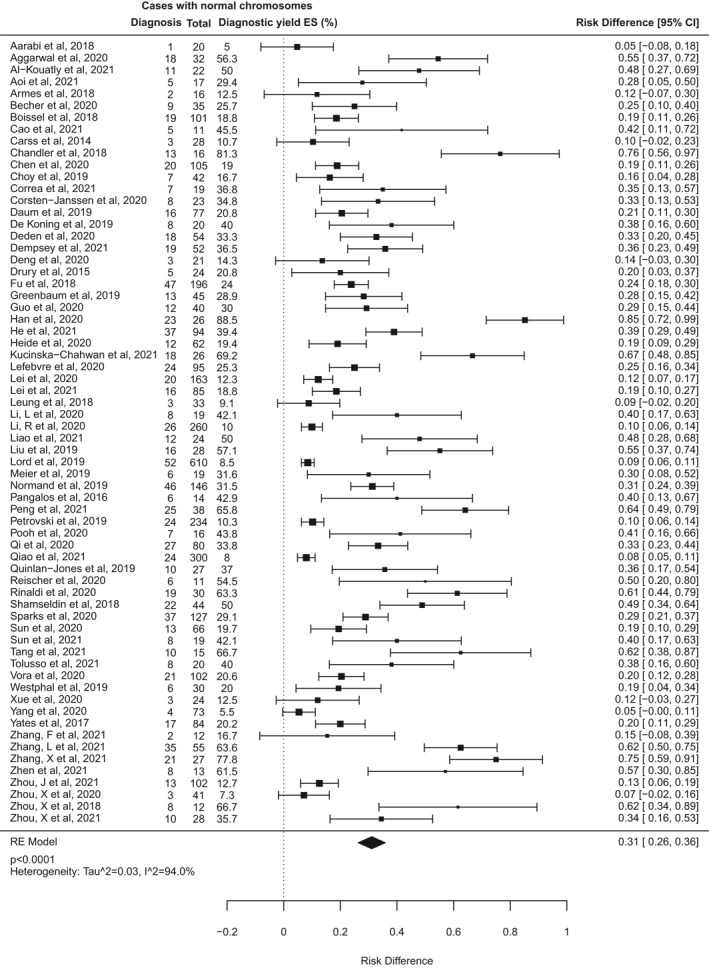
Forest plot showing individual and pooled incremental yield of prenatal exome sequencing (ES) over karyotype/chromosomal microarray from all 66 studies included in this review

The diagnostic yield of ES for fetal structural anomalies among the included studies ranged from 5% (95% CI −8 to 18%) to 89% (95% CI 72%–99%). The pooled incremental yield of ES over CMA/karyotype from all studies was 31% (95% CI 26%–36%, *p* < 0.0001), illustrated in the forest plot (Figure 3). Between‐study heterogeneity was high, indicated by an I^2^ statistic of 94% (*Q* = 682, *p* < 0.001). Testing for publication bias showed significant funnel plot asymmetry (*z* = 5.6, *p* < 0.0001 by Egger's test; supporting information S1), indicating possible publication bias.

### 3.3 Effect of case selection on diagnostic yield

**FIGURE 4:**
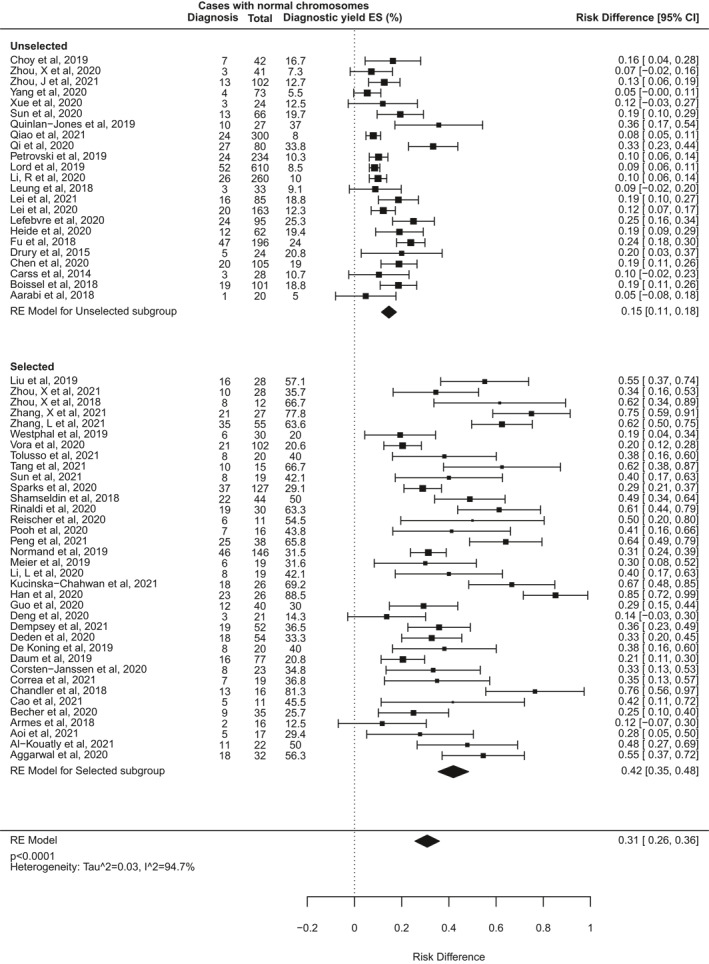
Forest plot showing individual and pooled incremental yield of prenatal exome sequencing (ES) over karyotype/chromosomal microarray analysed by pre‐test case selection criteria: “unselected” cohorts underwent no prior review to determine clinical features indicating higher likelihood of monogenic aetiology; “selected” cohorts underwent pre‐test expert review to select fetuses with clinical features indicating higher likelihood of monogenic disorder or to rule out non‐genetic conditions

Some of the heterogeneity between included studies may be due to differing approaches to case selection. Some studies included fetuses with structural anomalies without any prior review of the clinical features to ascertain risk of a monogenic aetiology, whereas others selected only pregnancies where the pattern of fetal anomalies was highly suggestive for a monogenic disorder (as determined by multidisciplinary teams [MDTs] usually including clinical genetics specialists). Seven studies were excluded from this analysis because it was not possible to determine how the cases were selected, or because mixed selection criteria were used. The other 59 studies were classified as selected or unselected cohorts (Table 2) for a subgroup analysis (Figure 4). This demonstrated that the pooled incremental diagnostic yield of ES for selected cohorts was 42% (95% CI 35%–48%), which is significantly higher than that in unselected cohorts (15%, 95% CI 11%–18%; *p* < 0.0001).

**TABLE 2:** Effect of case selection on incremental diagnostic yield of ES over CMA

Study	Number of probands with normal CMA	Diagnostic yield (%)
Unselected fetuses: NO prior review/selection for features indicating higher likelihood of monogenic disease		
Quinlan‐Jones et al, 2019	27	10/27 (37%)
Qi et al, 2020	80	27/80 (33.8%)
Lefebvre et al, 2020	95	24/95 (25.3%)
Fu et al, 2018	196	47/196 (24%)
Drury et al, 2015	24	5/24 (20.8%)
Sun et al, 2020	66	13/66 (19.7%)
Heide et al, 2020	62	12/62 (19.4%)
Chen et al, 2020	105	20/105 (19%)
Boissel et al, 2018	101	19/101 (18.8%)
Lei et al, 2021	85	16/85 (18.8%)
Choy et al, 2019	42	7/42 (16.7%)
Zhou, J et al, 2021	102	13/102 (12.7%)
Xue et al, 2020	24	3/24 (12.5%)
Lei et al, 2020	163	20/163 (12.3%)
Carss et al, 2014	28	3/28 (10.7%)
Petrovski et al, 2019	234	24/234 (10.3%)
Li, R et al, 2020	260	26/260 (10%)
Leung et al, 2018	33	3/33 (9.1%)
Lord et al, 2019	610	52/610 (8.5%)
Qiao et al, 2021	300	24/300 (8%)
Zhou, X et al, 2020	41	3/41 (7.3%)
Yang et al, 2020	73	4/73 (5.5%)
Aarabi et al, 2018	20	1/20 (5%)
**Pooled estimated diagnostic yield for unselected subgroup**	**2771**	**15% [95% CI = 11%–18%] *p* < 0.0001**
Selected fetuses: Prior expert review/selection for clinical features indicating higher likelihood of monogenic disorder or ruling out non‐genetic conditions		
Han et al, 2020	26	23/26 (88.5%)
Chandler et al, 2018	16	13/16 (81.3%)
Zhang, X et al, 2021	27	21/27 (77.8%)
Kucińska‐Chahwan et al, 2021	26	18/26 (69.2%)
Tang et al, 2021	15	10/15 (66.7%)
Zhou, X et al, 2018	12	8/12 (66.7%)
Peng et al, 2021	38	25/38 (65.8%)
Zhang, L et al, 2021	55	35/55 (63.6%)
Rinaldi et al, 2020	30	19/30 (63.3%)
Liu et al, 2019	28	16/28 (57.1%)
Aggarwal et al, 2020	32	18/32 (56.3%)
Reischer et al, 2020	11	6/11 (54.5%)
Al‐Kouatly et al, 2021	22	11/22 (50%)
Shamseldin et al, 2018	44	22/44 (50%)
Cao et al, 2021	11	5/11 (45.5%)
Pooh et al, 2020	16	7/16 (43.8%)
Li, L et al, 2020	19	8/19 (42.1%)
Sun et al, 2021	19	8/19 (42.1%)
De Koning et al, 2019	20	8/20 (40%)
Tolusso et al, 2021	20	8/20 (40%)
Correa et al, 2021	19	7/19 (36.8%)
Dempsey et al, 2021	52	19/52 (36.5%)
Zhou, X et al, 2021	28	10/28 (35.7%)
Corsten‐Janssen et al, 2020	23	8/23 (34.8%)
Deden et al, 2020	54	18/54 (33.3%)
Meier et al, 2019	19	6/19 (31.6%)
Normand et al, 2019	146	46/146 (31.5%)
Guo et al, 2020	40	12/40 (30%)
Aoi et al, 2021	17	5/17 (29.4%)
Sparks et al, 2020	127	37/127 (29.1%)
Becher et al, 2020	35	9/35 (25.7%)
Daum et al, 2019	77	16/77 (20.8%)
Vora et al, 2020	102	21/102 (20.6%)
Westphal et al, 2019	30	6/30 (20%)
Deng et al, 2020	21	3/21 (14.3%)
Armes et al, 2018	16	2/16 (12.5%)
**Pooled estimated diagnostic yield for selected subgroup**	**1293**	**42% [95% CI = 35%–48%] *p* < 0.0001**

*Note*: The bold and underlined formatting was just for emphasis in defining the two sub‐groups.

Abbreviations: CMA, chromosomal microarray; ES, exome sequencing.

### 3.4 Effect of fetal phenotypic group on diagnostic yield

Further heterogeneity may arise from the varied types of fetal structural anomaly included in different studies. To investigate this, a second subgroup analysis was performed for 42 studies that reported cases disaggregated by phenotypic group, and where it was possible to distinguish isolated system anomalies from multisystem anomalies (Figure 5). Data from at least 3 studies were available for fetuses in each of 13 phenotypic subgroups based on affected body system (Table 3). The number of fetuses analysed in each subgroup varied widely, from seven cases with isolated abdominal wall abnormalities, to 773 cases with isolated cardiac abnormalities. The subgroups for multisystem anomalies, isolated skeletal anomalies, and isolated brain or central nervous system (CNS) anomalies were the next largest, with over 400 cases each. The subgroup analysis found that the incremental diagnostic yield of ES differed significantly between different phenotypic groups (*I* ^2^ 96.4%, *Q* = 177, *p* < 0.0001). Table 3 details the pooled estimated incremental diagnostic yield for each subgroup, from highest to lowest. The highest diagnostic yields occurred in fetuses with skeletal abnormalities (53% [95% CI 42%–63%], *p* < 0.0001), neuromuscular abnormalities, usually presenting as fetal akinesia deformation sequence (FADS), (37% [20%–54%], *p* < 0.0001), and abnormalities in multiple systems (29% [22%–35%], *p* < 0.0001). By contrast, the lowest rates of ES diagnoses occurred in fetuses with isolated increased nuchal translucency (NT; 2% [0%–5%], *p* = 0.04), and those with gastrointestinal abnormalities (2% [−4 to 8%], *p* = 0.5). There were no diagnoses from ES in 7 fetuses with abdominal wall defects and 38 fetuses with chest abnormalities.

FIGURE 5Forest plot showing individual and pooled incremental yield of prenatal exome sequencing (ES) over karyotype/chromosomal microarray analysed by phenotypic sub‐group in studies where fetuses with anomalies confined to a single body system were distinguishable from those with multi‐system anomalies

**Figure:**
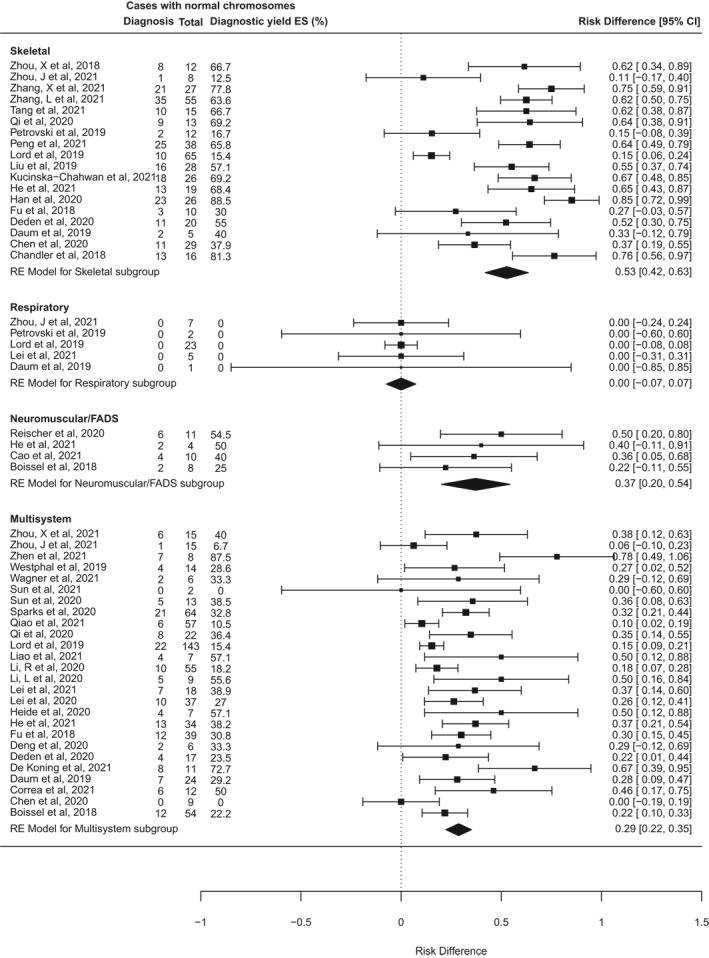

**Figure:**
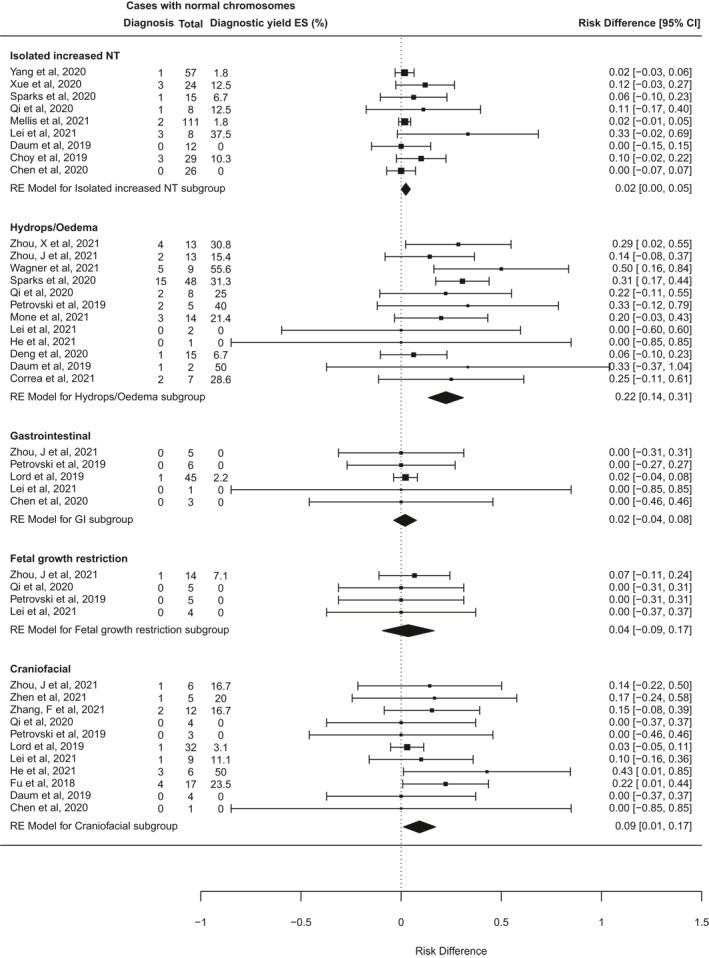

**Figure:**
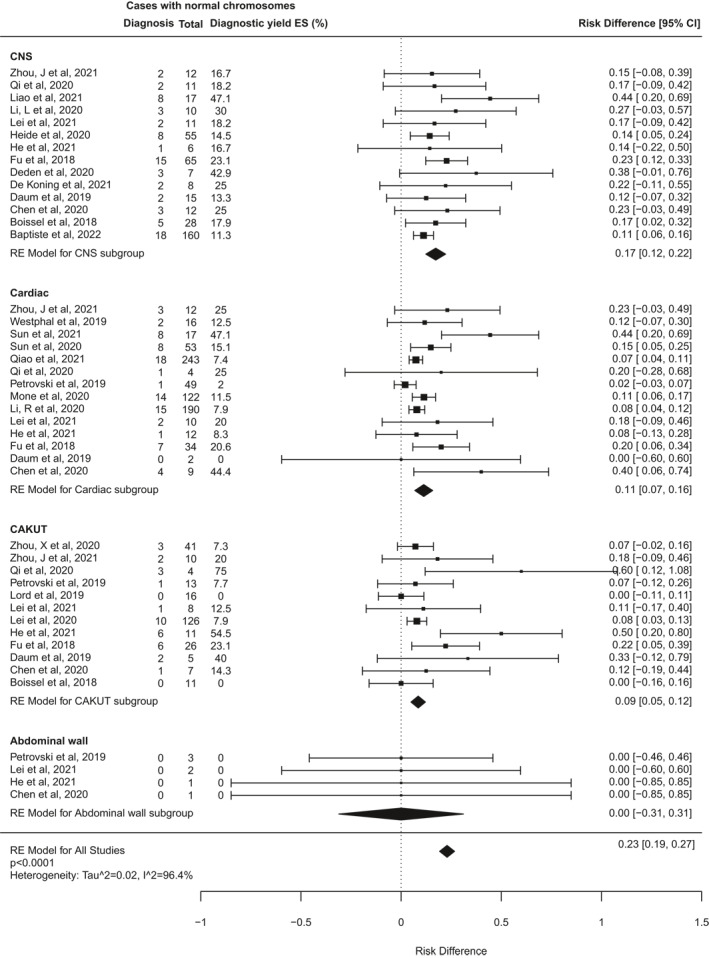

**TABLE 3:** Pooled effect size for incremental diagnostic yield of ES over CMA in different phenotypic groups

Phenotypic group	Cases analysed	Pooled estimated diagnostic yield [95% CI], *p*‐value
Skeletal	424	53% [42%–63%], *p* < 0.0001
Neuromuscular/Fetal akinesia deformation sequence (FADS)	33	37% [20%–54%], *p* < 0.0001
Multisystem	698	29% [22%–35%], *p* < 0.0001
Hydrops/Oedema	137	22% [14%–31%], *p* < 0.0001
Central nervous system (CNS)	417	17% [12%–22%], *p* < 0.0001
Cardiac	773	11% [7%–16%], *p* < 0.0001
Craniofacial	99	9% [1%–17%], *p* = 0.02
Congenital anomalies of kidneys and urinary tract (CAKUT)	278	9% [5%–12%], *p* < 0.0001
Fetal growth restriction	28	4% [−9 to 17%], *p* = 0.59
Isolated increased nuchal translucency (NT)	290	2% [0%–5%], *p* = 0.04
Gastrointestinal	60	2% [−4 to 8%], *p* = 0.5
Respiratory/Chest	38	0 [−7 to 7%], *p* = 1
Abdominal wall	7	0 [−31% to 31%], *p* = 1

*Note*: Phenotypic groups refer to fetuses with one or more anomalies in a single body system. Fetuses with anomalies in more than one system are classified as ‘Multisystem’. The ‘isolated increased NT’ group contains a mixture of (i) cases with isolated increased NT at presentation where it was unspecified whether additional anomalies developed later in pregnancy, and (ii) cases where isolated increased NT remained isolated throughout pregnancy. The bold and underlined formatting was just for emphasis in defining the two sub‐groups.

Abbreviations: CMA, chromosomal microarray; ES, exome sequencing.

### 3.5 Impact of prenatal ES on decision‐making and clinical management

Only seven reports from six studies explicitly assessed the impact of prenatal ES on clinical management. These studies reported that ES results influenced at least one of (i) decisions about termination of pregnancy, (ii) pre‐ or perinatal management, (iii) future reproductive planning, or (iv) counselling on recurrence risk in 26%–79% of cases.^8^ ^,^ ^26^ ^,^ ^52^ ^,^ ^62^ ^,^ ^73^ ^,^ ^74^ ^,^ ^82^

## 4 DISCUSSION

The findings of our review underscore the value of applying ES in the prenatal context. Meta‐analysis results show an incremental diagnostic yield of 31% from ES over CMA/karyotype alone for prenatal diagnosis of fetal structural anomalies. This diagnostic yield could be considered a conservative estimate because we included only definite diagnoses (variants classified as pathogenic/likely pathogenic *and* deemed to be causing the fetal phenotype) in our definition of diagnostic yield. Variants reported as ‘possibly diagnostic’, ‘probably diagnostic’, or ‘potentially relevant’ were not included in our analysis but may have been re‐classified as diagnostic after further study. For example, one study of 19 fetuses with severe multiple anomalies of suspected genetic aetiology found a diagnostic yield of 31.6% when strictly using ACMG guidelines, but this rose to 63% when including variants not reportable by ACMG criteria but which were plausible based on animal models, in silico prediction tools or functional studies.^68^ Other studies have also shown potentially increased diagnostic yields when probable diagnoses are included.^30^ ^,^ ^71^

In this way, diagnostic yields can increase over time, as has been previously documented in large postnatal ES studies such as the UK Deciphering Developmental Disorders (DDD) study, where iterative reanalysis was performed after several years.^89^ This results from improved bioinformatics methodologies, newly discovered gene‐disease associations and, in the case of prenatal ES, evolving knowledge of fetal presentations of known disorders. For example, in one study from our review, Petrovski et al. reported four variants which were re‐classified (from bioinformatic signatures) to diagnostic variants over the course of the study period in light of new published evidence or additional phenotypes emerging postnatally.^13^

Our second key finding is that the diagnostic yield of ES for fetal anomalies is higher when cases are pre‐selected for likelihood of a monogenic aetiology by MDTs with expertise in genetics. This is intuitive but, as prenatal ES is increasingly implemented in clinical diagnostic settings, it highlights the value of pre‐test review and MDT collaboration to direct testing where it is most likely to yield a diagnosis. Furthermore, our findings demonstrate that diagnostic yield varies significantly between phenotypic groups: the highest yield observed was for skeletal dysplasias (53%) and the lowest yields were for fetuses with isolated increased NT (2%) and isolated gastrointestinal anomalies (2%).

This understanding of likely diagnostic yield for different fetal presentations may inform clinical guidelines on offering prenatal ES, however our findings should be interpreted with care. Classification of cases by phenotype and comparison across studies is challenging because of different approaches to the classification (e.g. whether ‘soft markers’ are classed as separate abnormalities, and whether cases were categorised strictly based on isolated system anomalies or based on the system that best describes the *predominant* phenotype in the fetus). Moreover, fetal phenotypes evolve as organ development progresses, so classification of the same case may differ depending on the gestation at assessment.^23^ Further research is needed to clarify the diagnostic utility of prenatal ES for isolated anomalies in systems that have been less thoroughly investigated to date. For example, in this review, for fetuses with chest (respiratory) abnormalities, fetal growth restriction, gastrointestinal anomalies and abdominal wall defects, the sample sizes were too small to reach statistical significance in estimates of pooled diagnostic yield.

Studies examining other aspects of clinical utility such as impact on clinical management and parental decision‐making will be an important complement to this,^90^ however we found only six to include in our review.^8^ ^,^ ^26^ ^,^ ^52^ ^,^ ^62^ ^,^ ^73^ ^,^ ^74^ ^,^ ^82^ Three of these studies found that negative as well as diagnostic results impacted upon parental and clinical decision‐making,^73^ ^,^ ^74^ ^,^ ^82^ leading Deden et al. to conclude that ‘the efficacy of prenatal [ES] should be evaluated by more variables than diagnostic yield alone.’^73^ This is an area where the need for further research is pressing, including more studies exploring the perspectives of parents undergoing prenatal ES to investigate what personal utility they derive from it.

### 4.1 Strengths and limitations

This systematic review is the most detailed and comprehensive in this area to date. It included 72 reports of 66 studies, many of which were published in the last 12 months. The reporting of research in the included studies was generally of high quality with a standardised approach to reporting which made it easier to compare study characteristics, and we were able to perform meta‐analyses for a range of phenotypic groups as well as overall. There are however signs of possible publication bias, as indicated by funnel plot asymmetry showing a paucity of published studies with small sample size and low diagnostic yield (supporting information S1). This could reflect publication bias or indeed may represent a true relationship between small sample size and higher diagnostic yield: among the studies included in this review, smaller cohorts were often highly selected (usually after review by clinical genetics specialists) for cases where the clinical presentation suggested a monogenic aetiology. The higher a priori likelihood of a monogenic disorder among such cases translates to higher diagnostic yields, whereas the largest studies to date have been unselected cohorts, including fetuses with isolated and minor abnormalities and anomalies associated with multifactorial aetiology, as well as those more likely to have a monogenic disorder.

The main limitation of our review was the high degree of heterogeneity between included studies, limiting the precision of comparisons. On performing subgroup analyses to investigate the effect on ES diagnostic yield of case selection criteria or fetal phenotypic group, there remained substantial heterogeneity within many of the subgroups as well as between them, which suggests that neither of these factors alone fully explain the observed heterogeneity amongst the studies. Studies also varied in sample size, sequencing and analysis approach (gene agnostic or targeted), approach to variant interpretation, and whether or not interpretation considered postnatal/post‐mortem phenotype information in addition to prenatal phenotypes. All of these factors are likely to influence the diagnostic yield. For example, even when adhering to standardised ACMG variant interpretation guidelines, there may be some subjectivity in how the guidelines are applied and how it is judged whether a variant explains the fetal phenotype. Studies done in a purely research setting without reporting results back to families may have interpreted variants with candidate causality more generously, leading to higher reported diagnostic yields than in studies that used more conservative reporting standards. In addition, for the phenotype subgroup analysis we only included studies that disaggregated cases by phenotypic group and distinguished cases with a single affected body system from those with multiple system anomalies. This resulted in the exclusion of 24/66 studies from this subgroup analysis. Manual inspection of the raw phenotype data from these studies could enable extraction and classification of more cases in their phenotypic groups and allow a more granular analysis of the incremental yield of ES in different phenotypic groups, as has been reported recently for congenital heart disease, fetal hydrops, and isolated increased NT respectively.^24^ ^,^ ^25^ ^,^ ^91^

## 5 CONCLUSION

Prenatal ES is a powerful tool to improve the diagnosis of monogenic conditions in fetuses with structural abnormalities. However, it also presents challenges for interpreting variants in the face of incomplete and non‐specific fetal phenotypes while also aiming to minimise identification of VUS and incidental findings. In ongoing pregnancies, results must be returned quickly if they are to inform decisions on termination of pregnancy or prenatal management. Prenatal ES is rapidly being adopted in many clinical settings and its successful implementation will require robust laboratory infrastructure and bioinformatics pipelines, expert parental counselling, multidisciplinary collaboration between fetal medicine and clinical and laboratory genetics specialists to offer and interpret tests appropriately, and clear guidelines to ensure equity of access. The need to sequence fetus‐parent trios in parallel to minimise turn‐around times means that ES remains relatively costly, so in many health systems it may be necessary to triage cases to allocate finite resources. Further research is needed on the clinical impact of prenatal ES, to better understand which pregnancies benefit most and how to prioritise potential cases in a way that maximises benefit to families, and is efficient for healthcare systems.

## CONFLICT OF INTEREST

None of the authors have any conflict of interests to declare.

## ETHICS STATEMENT

This work is a systematic review of literature and no ethical committee approval was required.

## Supporting information

Supplementary Material 1

## Data Availability

Data sharing is not applicable to this article as no new data were created or analysed in this study. The data that support the findings of this study are derived from the cited publicly available published studies included in the review.
