# Supplementary material for: Diagnostic yield of exome sequencing for prenatal diagnosis of fetal structural anomalies: A systematic review and meta‐analysis
Source: Prenat Diagn. 2022 May 7;42(6):662–85. doi: 10.1002/pd.6115 (PMC9325531; doi:10.1002/pd.6115)
Supplement: Supplementary file 1 — Supplementary Material 1 [file PD-42-662-s001.docx]

**Supplementary Information**

**1. Search strategy**

Keywords: (Fetal OR Fetus OR Foetal OR Foetus OR Prenatal OR Antenatal) OR MeSH terms: (Prenatal Diagnosis OR Fetal diseases OR Fetal development)

AND

Keywords: (Exome sequenc* OR WES OR Whole genome sequenc* OR WGS OR Genome-wide) OR MeSH terms: (Whole Exome Sequencing OR Exome OR Whole Genome Sequencing OR Genome, Human OR Sequence Analysis, DNA)

where * indicates right-hand truncation with unlimited variations, in order to include sequence/sequences/sequencing etc.

**1a) Search strategy as conducted in Cochrane library**

ID Search

#1 MeSH descriptor: [Prenatal Diagnosis] explode all trees

#2 MeSH descriptor: [Fetal Diseases] explode all trees

#3 MeSH descriptor: [Fetal Development] explode all trees

#4 #1 or #2 or #3

#5 Fetal OR Fetus OR Foetal OR Foetus OR Prenatal OR Antenatal (Word variations have been searched)

#6 #4 or #5

#7 MeSH descriptor: [Sequence Analysis, DNA] explode all trees

#8 MeSH descriptor: [Exome] explode all trees

#9 MeSH descriptor: [Whole Exome Sequencing] explode all trees

#10 MeSH descriptor: [Genome] explode all trees

#11 MeSH descriptor: [Whole Genome Sequencing] explode all trees

#12 #7 or #8 or #9 or #10 or #11

#13 Exome sequencing OR WES OR Whole genome sequencing OR WGS OR Genome-wide (Word variations have been searched)

#14 #12 or #13

#15 #6 and #14 with Cochrane Library publication date Between Jan 2010 and Dec 2021

**1b) Search strategy as conducted in Web of Science**

<https://www.webofscience.com/wos/woscc/summary/90e0b6e0-9e08-46b4-9b07-12c6effbeac6-0d484728/relevance/1>

Search of CORE COLLECTION

ID Search (number of results)

#1 TS = (Fetal OR Fetus OR Foetal OR Foetus OR Prenatal OR Antenatal) ([444,735](https://www-webofscience-com.libproxy.ucl.ac.uk/wos/woscc/summary/985c75e5-dddf-4d6c-877d-0fdeb438e7be-0d483db7/relevance/1))

#2 TS = ("Exome sequenc*" OR WES OR "Whole genome sequenc*" OR WGS OR Genome-wide) ([179,753](https://www-webofscience-com.libproxy.ucl.ac.uk/wos/woscc/summary/29ffbf15-3cb4-4a81-81c4-7018d82ca96c-0d483f75/relevance/1))

#3 #1 AND #2 ([3,381](https://www-webofscience-com.libproxy.ucl.ac.uk/wos/woscc/summary/6d65dd0d-9392-431e-9785-1135d502c3c7-0d48402f/relevance/1))

#4 #3 ([3,125](https://www-webofscience-com.libproxy.ucl.ac.uk/wos/woscc/summary/9732351d-722f-4914-b2f8-979e1e1b559a-0d4841cc/relevance/1))

**1c) Search strategy as conducted in Embase**

Database: Embase <1980 to 2021 Week 40>

Search Strategy:

ID Search (number of results)

--------------------------------------------------------------------------------

1 Prenatal Diagnosis/ (57350)

2 Fetal Diseases/ or Fetal Development/ (27290)

3 1 or 2 (81998)

4 (Fetal or Fetus or Foetal or Foetus or Prenatal or Antenatal).mp. [mp=title, abstract, heading word, drug trade name, original title, device manufacturer, drug manufacturer, device trade name, keyword heading word, floating subheading word, candidate term word] (644937)

5 3 or 4 (644937)

6 Sequence Analysis, DNA/ or Exome/ or Whole Exome Sequencing/ (54297)

7 Whole Genome Sequencing/ or Genome, Human/ (44107)

8 6 or 7 (95133)

9 (Exome sequenc* or WES or Whole genome sequenc* or WGS or Genome-wide).mp. [mp=title, abstract, heading word, drug trade name, original title, device manufacturer, drug manufacturer, device trade name, keyword heading word, floating subheading word, candidate term word] (209570)

10 8 or 9 (234857)

11 5 and 10 (6385)

12 limit 11 to yr="2010 -Current" (6000)

***************************

**1d) Search strategy as conducted in MEDLINE**

Database: Ovid MEDLINE(R) ALL <1946 to October 14, 2021>

Search Strategy:

ID Search (number of results)

--------------------------------------------------------------------------------

1 Prenatal Diagnosis/ (38450)

2 Fetal Diseases/ or Fetal Development/ (38257)

3 1 or 2 (70003)

4 (Fetal or Fetus or Foetal or Foetus or Prenatal or Antenatal).mp. [mp=title, abstract, original title, name of substance word, subject heading word, floating sub-heading word, keyword heading word, organism supplementary concept word, protocol supplementary concept word, rare disease supplementary concept word, unique identifier, synonyms] (561592)

5 3 or 4 (561592)

6 Sequence Analysis, DNA/ or Exome/ or Whole Exome Sequencing/ (174173)

7 Whole Genome Sequencing/ or Genome, Human/ (36888)

8 6 or 7 (205536)

9 (Exome sequenc* or WES or Whole genome sequenc* or WGS or Genome-wide).mp. [mp=title, abstract, original title, name of substance word, subject heading word, floating sub-heading word, keyword heading word, organism supplementary concept word, protocol supplementary concept word, rare disease supplementary concept word, unique identifier, synonyms] (146223)

10 8 or 9 (320391)

11 5 and 10 (5160)

12 limit 11 to yr="2010 -Current" (3887)

***************************

**
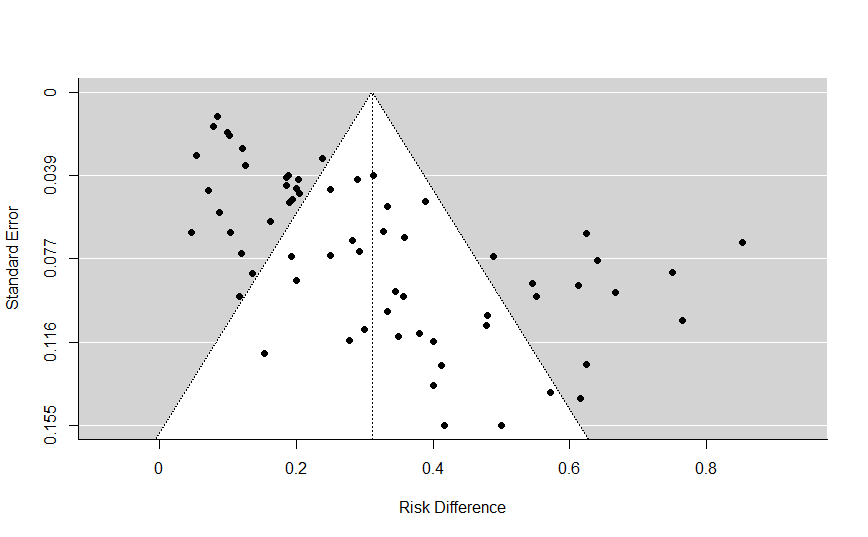
**

**Figure S2.** Funnel plot of included studies, demonstrating each study’s incremental diagnostic yield of exome sequencing (expressed as risk difference) against its standard error. Standard error is inversely proportional to sample size so larger studies are represented in the upper part of the plot, with smaller studies below. The plot demonstrates a paucity of studies with small sample size and low diagnostic yield.
